# It's a kind of magic—what self-reports can reveal about the phenomenology of insight problem solving

**DOI:** 10.3389/fpsyg.2014.01408

**Published:** 2014-12-08

**Authors:** Amory H. Danek, Thomas Fraps, Albrecht von Müller, Benedikt Grothe, Michael Öllinger

**Affiliations:** ^1^Division of Neurobiology, Department Biology II, Ludwig-Maximilians-Universität MünchenMunich, Germany; ^2^Trick 17 Magic ConceptsMunich, Germany; ^3^Parmenides FoundationMunich, Germany; ^4^Department Psychology, Ludwig-Maximilians-Universität MünchenMunich, Germany

**Keywords:** insight, problem solving, magic, Aha! experience, impasse

## Abstract

Magic tricks usually remain a mystery to the observer. For the sake of science, we offered participants the opportunity to discover the magician's secret method by repeatedly presenting the same trick and asking them to find out how the trick worked. In the context of insightful problem solving, the present work investigated the emotions that participants experience upon solving a magic trick. We assumed that these emotions form the typical “Aha! experience” that accompanies insightful solutions to difficult problems. We aimed to show that Aha! experiences can be triggered by magic tricks and to systematically explore the phenomenology of the Aha! experience by breaking it down into five previously postulated dimensions. 34 video clips of different magic tricks were presented up to three times to 50 participants who had to find out how the trick was accomplished, and to indicate whether they had experienced an Aha! during the solving process. Participants then performed a comprehensive quantitative and qualitative assessment of their Aha! experiences which was repeated after 14 days to control for its reliability. 41% of all suggested solutions were accompanied by an Aha! experience. The quantitative assessment remained stable across time in all five dimensions. Happiness was rated as the most important dimension. This primacy of positive emotions was also reflected in participants' qualitative self-reports which contained more emotional than cognitive aspects. Implementing magic tricks as problem solving task, we could show that strong Aha! experiences can be triggered if a trick is solved. We could at least partially capture the phenomenology of Aha! by identifying one prevailing aspect (positive emotions), a new aspect (release of tension upon gaining insight into a magic trick) and one less important aspect (impasse).

## Introduction

Sometimes, the solution to a difficult problem pops into mind suddenly (Davidson, 1995) and unexpectedly (Metcalfe, 1986). Ever since the Gestalt psychologists (Köhler, 1921; Duncker, 1945; Wertheimer, 1959) began to investigate problem solving, the phenomenon of insight has been of great interest to psychologists (Sternberg and Davidson, 1995). Insight is often reported to be accompanied by an affective response, the “Aha! experience” (e.g., Gick and Lockhart, 1995). This is taken as the discriminative criterion to set it apart from analytic and gradual problem solving (Metcalfe, 1986; Evans, 2008).

Bühler provided the first reports about Aha! experiences, describing a moment “in which suddenly, the lights come on” (translated from Bühler, 1907, p. 341). Traditionally, the Aha! has been regarded as an interesting epiphenomenon of insight (e.g., Ormerod et al., 2002) or even the defining feature of insight (Kaplan and Simon, 1990; Gick and Lockhart, 1995) that defies closer empirical inquiry due to its subjective nature. But the recent interest in possible neural correlates of insight has led to a surge in studies that presuppose the subjective Aha! experience to be the clearest observable aspect of insight (Jung-Beeman et al., 2004), at least until a better behavioral or even neural marker of the occurrence of insight is found. Consequently, many of these studies rely on problem solvers' subjective reports about the occurrence of an Aha! experience to classify a solution as insightful and to distinguish it from solutions without insight (Bowden et al., 2005; Aziz-Zadeh et al., 2009). However, unsolved questions remain, especially with regard to methodology.

The methodological difficulties inherent to insight research have been recognized and discussed in the field (Davidson, 1995, 2003; Chronicle et al., 2004; Ash et al., 2009; Öllinger and Knoblich, 2009). The debate has revolved around the question of whether there are specific insight problems and if so, what defines them. In our opinion, insight problems “*per se*” don't exist (see Öllinger et al., 2013). Any problem can be solved with or without insight, depending on the problem solver's prior knowledge. Of course, some problems are more likely to be solved with insight, like the famous nine-dot problem (Scheerer, 1963). When prior knowledge leads to a biased initial problem representation (Ohlsson, 1992), a representational change is necessary to overcome self-imposed constraints resulting in an enhanced problem representation that might be appropriate to solve the problem. Unfortunately, the underlying processes of representational changes are opaque. To deal with this problem, a common approach is to ask solvers whether they experienced any changes before a solution occurred. A related unsolved problem is how to assess the occurrence of insight. A well-known observation reported by a vast number of participants is the feeling of Aha! that accompanies the moment of insight. Consequently, each solution can be classified by asking participants whether they had or had not experienced an Aha! moment. Bowden and colleagues advocate the use of such self-reports (Bowden et al., 2005) instead of defining a priori what an insight problem is or not. This means, participants are asked after each solution to report on their subjective experience of insight, indicated by the Aha! experience. The problem solver, not the experimenter, decides whether insight has occurred or not.

We aim at elaborating and differentiating the phenomenological experience before an insight solution occurs—the precondition to identifying reliable markers that demarcate insight from non-insight problem solving and for properly understanding the cognitive and neural processes underlying insight problem solving.

We believe that the self-report approach could help to advance insight research, if it is possible to show that such reports are reliable measures, e.g. that they can be repeated over time. We therefore asked whether participants would be able to remember their self-reports after a long delay (2 weeks). Of course, the Aha! experience itself cannot be repeated, only the reports on it. If the Aha! experience is indeed such a strong affective experience, we expect people to remember it clearly. This should be reflected in similar ratings across time, when asked to think back to their Aha! experiences. Another reason to expect a high reliability is the fact that self-reports have already been successfully adopted in other studies as a tool to differentiate insight from non-insight (Sandkühler and Bhattacharya, 2008; Sheth et al., 2009; Subramaniam et al., 2009). It was even possible to reveal different neural activity underlying insight and non-insight solutions, for example, Kounios et al. (2006) analyzed a time interval of 2 s prior to problem presentation and found differences in neural activity (both in the EEG and in the fMRI signal) predicting whether the following problem would be solved with insight (Aha! reported) or without insight (Aha! not reported). Investigating the memorial advantage of insight, we have also used participants' self-reports and found that solutions that had been classified as insightful were remembered better in comparison to non-insight solutions (Danek et al., 2013). In the present work, we adopted Bowden's approach (2005) to determine the occurrence of insight and combined this approach with an a priori selection of a task (magic tricks) that is likely to trigger misleading initial problem representations.

Despite its successful use as a solution type classification criterion and its importance for the interpretation of almost all neuroscientific studies on insight problem solving, the phenomenology of the Aha! experience, as far as we know, has not been investigated in more detail. One hindrance is the methodological difficulty of its assessment (introspective judgments about the occurrence of Aha!), another one might be conceptual problems (what defines an Aha! experience?). So far, there is no general and explicit agreement on a definition of this concept. The common denominator is that an Aha! occurs if a solution suddenly pops into mind. Other aspects like a feeling of surprise, certainty that the solution is correct or a gestalt-like quality of the solution are stressed or disregarded to various degrees across studies (Ohlsson, 1992; Bowden et al., 2005; Sandkühler and Bhattacharya, 2008). The theoretical assumption that prior impasse is a necessary precondition for Aha! experiences to occur (Ohlsson, 1992; Knoblich et al., 2001; Jones, 2003; Öllinger et al., 2006) is taken up by some (e.g., Schooler et al., 1993; Sandkühler and Bhattacharya, 2008) and questioned by others (e.g., Bowden et al., 2005). The conceptual vagueness makes it very difficult to compare findings across studies, and thus it seems critical to further elucidate the phenomenology of this special experience (compare Gick's call for further research on the affective aspects of problem solving, Gick and Lockhart, 1995).

The aim of our study was to provide a detailed analysis of the Aha! experience during sudden moments of insight into magic tricks. We assumed a multidimensional model where the interplay of different dimensions establishes the Aha! experience and assessed the relative importance of the involved components quantitatively as well as qualitatively. As a basis for this assessment, we identified five dimensions of the Aha! experience that have been postulated previously:

1. Suddenness: That insightful solutions are experienced as very sudden was demonstrated by Metcalfe (Metcalfe, 1986; Metcalfe and Wiebe, 1987) who showed that although problem solvers are able to accurately judge their progress toward the solution (recorded as feeling-of-warmth ratings) for non-insight problems, they are unable to do so for insight problems. This finding was further confirmed by Davidson (1995).
2. Surprise: Based on introspection and informal observation, Gick and Lockhart (1995) suggested a division of the Aha! experience in two components: Surprise and suddenness. In their account, the surprise aspect can vary by strength and it can be accompanied by either positive (delight) or negative (chagrin) emotions. In order to disentangle surprise from these accompanying emotions, we decided to assess the emotional component separately, adding “happiness” as a new dimension.
3. Happiness: Because Gick and Lockhart (1995) proposed the emotional response to vary between the positive and negative pole, we used a scale with “unpleasant” and “pleasant” as two extremes. There is also anecdotical evidence for this dimension of the Aha! experience, for example Gruber (1995) who analyzed Darwin's notes from the time of his great discovery on 28th September, 1838 and from them, inferred “a state of elevated happiness” (1995, p. 425).
4. Impasse: Ohlsson postulated that prior impasse is a necessary precondition for Aha! experiences to occur (1992). An impasse is defined as a state of mind where problem solving behavior ceases (Ohlsson, 1992; Öllinger et al., 2008; Sandkühler and Bhattacharya, 2008). In an eye-movement study, Knoblich et al. (2001) demonstrated that for successful solvers of insight problems, the number of long fixation times (i.e., periods with few eye movements) increases throughout the problem solving process, with longest fixation times occurring in the last time interval before the solution. That is, before insight occurred, there was a phase without systematic eye-movement patterns. Their interpretation of such an “idling” phase was that more appropriate representations can be established that yield a new insight.
5. Certainty: Obviousness of solution, i.e., the certainty that an insightful solution is correct, was stressed as an additional aspect by Bowden and Jung-Beeman (2007). This “intuitive sense of success” related to insightful solutions is also often described in the context of scientific discoveries (Gick and Lockhart, 1995, p. 215).

Furthermore, we wanted to test Bowden's claim (2005) of the usefulness of subjective judgments. The differential assessment of the five dimensions was therefore repeated after 2 weeks to test their reliability. The present study addressed the following two hypotheses:

1. Multidimensionality: We assumed that the Aha! experience is a syndrome of well-defined characteristics and hypothesized that all five dimensions are equally important.
2. Reliability: We tested whether participants' assessment of their Aha! experiences would be stable across time and predicted that they would remember it well, resulting in similar ratings across time.

The present work focuses on the phenomenology of the Aha! experience. With the aim of triggering strong Aha! experiences, we used magic tricks as a problem solving task, assuming that gaining insight into a magic trick would lead to a strong affective response since the secret of a magic trick is typically extremely hard to find out. Further, we have shown previously that magic tricks are ideally suited to investigate insight because in order to discover the magicians' secret method, observers must overcome implicit constraints by restructuring their problem representation (Danek et al., 2014). This is a crucial aspect common to other insight problems, too (Ohlsson, 1992; Knoblich et al., 2001). We also claim that, in contrast to most classical insight problems, magic tricks are less artificially construed and are more “ecologically valid” stimuli in the sense that efforts to solve the tricks are naturally set in motion. When observing a magic trick, people are astonished and surprised and usually want to find out “how it was done,” i.e., how the magic effect was achieved. The magician deeply affects prior knowledge representations, by seemingly overturning them (e.g., a levitation effect that seems to defy gravity). Consequently, we assume that discovering the secret of a magic trick results also in an intense Aha! experience, comparable with finding the solution to classical paper-and-pencil tasks by insight. Most important, and this makes magic tricks superior to classical insight problems, it is possible to present a large number of consecutive problems that usually have a high attraction for the observer, so that we get much more data points than in classical studies that use only 1–5 insight problems (e.g., Fleck and Weisberg, 2004).

Previous research implementing magic tricks as stimuli supports our view: Parris and colleagues investigated the neural correlates of disbelief by contrasting video clips of magic tricks with other surprising video clips and found specific activity in the left dorsolateral prefrontal cortex (Parris et al., 2009). This shows that there is something special to magic tricks that goes beyond mere surprise—Parris et al. interpreted this activity as a detection mechanism for violations of causality which are the essence of most magic tricks. In another fMRI study to be published in the same Frontiers research topic (Danek et al., Submitted), we focused on these violations of causality with a new and larger set of magic tricks and could replicate some of Parris' findings. In addition, we found that the brain activity of the magician who had performed the tricks clearly differed from the brain activity of naïve observers. In contrast to lay participants, the trick apparently did not involve any causality violations for the magician himself (this can be compared to the scenario of a magician practicing his gestures in front of a mirror—and no magic effect takes place). In sum, observing a magic trick seemingly invalidates the spectator's implicit assumptions about what is possible in the world, and therefore leads to the strong desire to discover the secret behind the trick. If this is achieved, we assume that the typical Aha! experience will be triggered.

## Materials and methods

### Participants

Fifty students (mean age 24.4; 16 male) participated for 32€ in the study and were tested individually after giving informed consent. Two participants were excluded because they did not solve any of the presented tasks, resulting in a final sample size of 48. The experiment was approved by the Institutional Review Board (Ethics Committee) of the Department of Psychology, LMU Munich.

### Testing material

The testing material consisted of 37 (3 of them used for practice) video clips of magic tricks that had been performed by a professional magician (TF) and recorded in a standardized setting. The video clips that ranged from 6 to 80 s were presented on a 17” computer screen displayed by the Presentation® software version 12.1. The tricks covered a wide range of different magic effects (e.g., transposition, restoration, vanish) and methods (e.g., misdirection, gimmicks, optical illusions). The magic tricks were presented to participants as a problem solving task. See http://www.youtube.com/watch?v=3B6ZxNROuNw for a sample trick clip from our study. Stimulus development, a complete list of the tricks and the experimental rationale are described in further detail in another paper (Danek et al., 2014).

### Procedure

There were two separate testing sessions with exactly 14 days delay. In session 1, participants' task was to watch magic tricks and to find the secret method used by the magician to produce the magic effect. If a trick was solved, they had to indicate on a trial-by-trial basis whether they had experienced an Aha! during the solution. After completing all tricks, participants were asked to evaluate their Aha! experiences. 14 days later, participants were invited again for a second evaluation of their Aha! experiences, this time from memory. In addition, a recall of participants' solutions was conducted in session 2. The results of this recall do not contribute to the present research question and are thus reported elsewhere (Danek et al., 2013). Both sessions lasted about 2 h.

#### Session 1: magic tricks

Participants were seated in a distance of 80 cm in front of a computer screen. After filling in an informed consent, they were orally instructed by the experimenter to watch the video clips of magic tricks and think of a solution how the trick could work. If participants failed to solve the trick, the video clip was repeated up to two more times while solving attempts continued.

**Figure 1:**
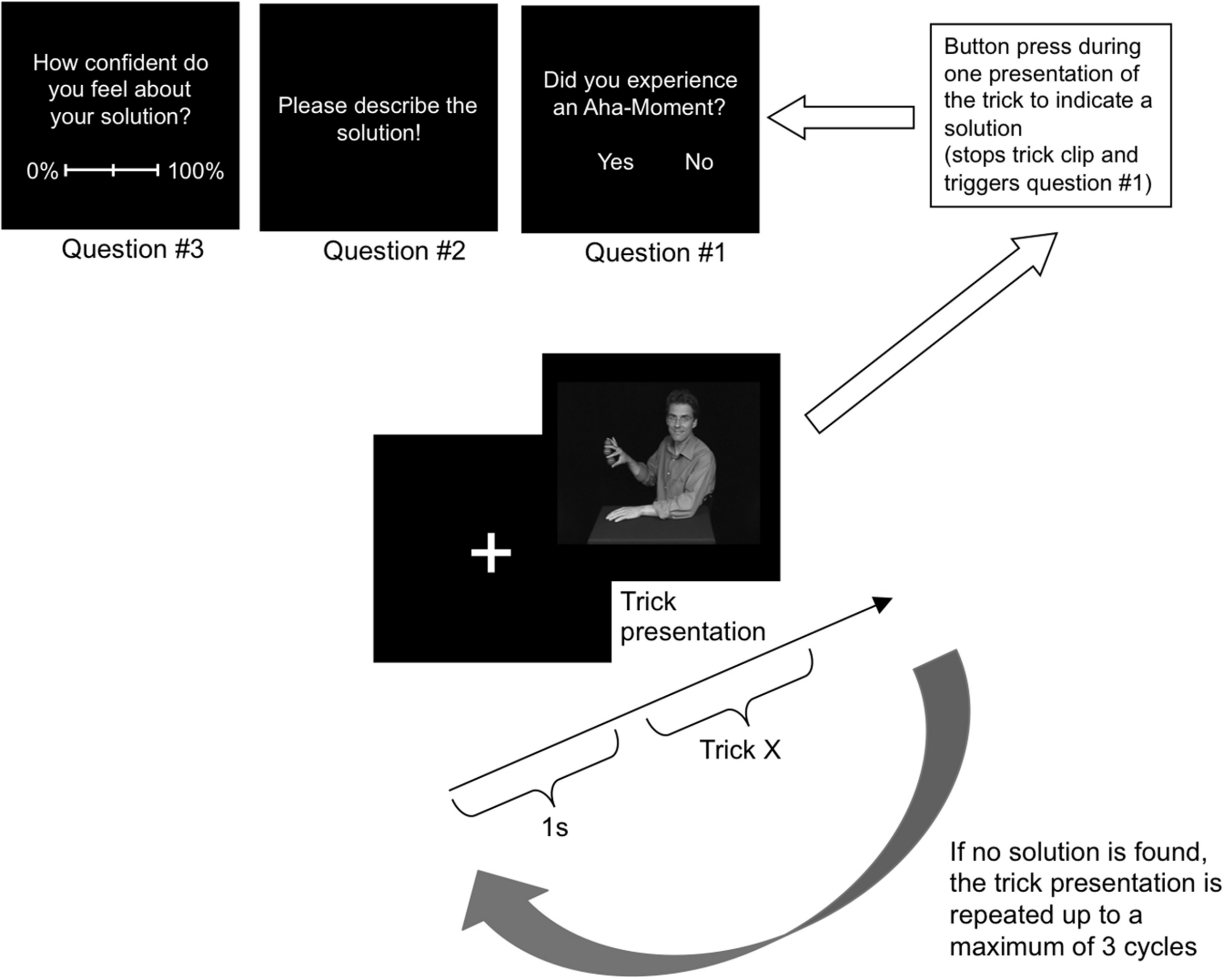
**Procedure of one trial**. Different phases and timing are displayed. Note that individual tricks vary in length.

As soon as they had found a potential solution, participants were required to press a button which stopped the video clip and ended the trial. A dialog with the following question appeared (all questions in German): Did you experience an Aha! moment? Participants indicated Yes or No with a mouse click. Subsequently, they were prompted to type in the solution on the keyboard and gave a certainty rating of how confident they felt about the correctness of their solution on a scale from 0 to 100%. Figure 1 shows the procedure of one trial.

Following Bowden and Jung-Beeman's approach (2007), participants categorized their solution experiences into insight (with Aha!) and non-insight (without Aha!) solutions. We used the following instruction for these judgments (adapted from Jung-Beeman et al., 2004): “We would like to know whether you experienced a feeling of insight when you solved a magic trick. A feeling of insight is a kind of “Aha!” characterized by suddenness and obviousness. Like an enlightenment. You are relatively confident that your solution is correct without having to check it. In contrast, you experienced no Aha! if the solution occurs to you slowly and stepwise, and if you need to check it by watching the clip once more. As an example, imagine a light bulb that is switched on all at once in contrast to slowly dimming it up. We ask for your subjective rating whether it felt like an Aha! experience or not, there is no right or wrong answer. Just follow your intuition.”

After three practice trials, the experiment started and for each participant, a randomized sequence of 34 magic tricks was presented.

#### Session 1: assessment of Aha! experience

Adopting a similar procedure from MacGregor and Cunningham (2008) who collected a global self-rating of insight after participants had worked on several different insight problems, we decided to conduct the comprehensive assessment after all tasks were completed. This procedure of asking participants to report their overall feeling of Aha! allowed us to collect the most basic, overarching characteristics of the insight experience, independent from individual fluctuations caused by differences between single problems (e.g., a very difficult trick in contrast to a less difficult one that might lead to less strong Aha! experiences). We used a two-fold approach:

- Self-report (qualitative): participants were given the opportunity to describe the thoughts and emotions that occurred while they gained insight into the working of a magic trick. This self-report was performed prior to the rating of importance to avoid possible transfer effects—so that participants could freely describe their actual Aha! experience without being influenced by the given dimensions.
- Rating of importance (quantitative): five previously postulated dimensions were subjected to a rating of importance by participants (compare Sandkühler and Bhattacharya, 2008).

***Session 1: self-report***. After completing all 34 magic tricks, participants were asked to give introspective self-reports (“Think back to the Aha! moments that you had during the experiment. For you, how does an Aha! moment feel like? Please describe it in your own words!”). It was stressed that the self-reports should refer to Aha! solutions only, not to the other solutions which participants had classified as non-insightful. Participants used the keyboard to type in their descriptions. There was no time limit for this task.

**Figure 2:**
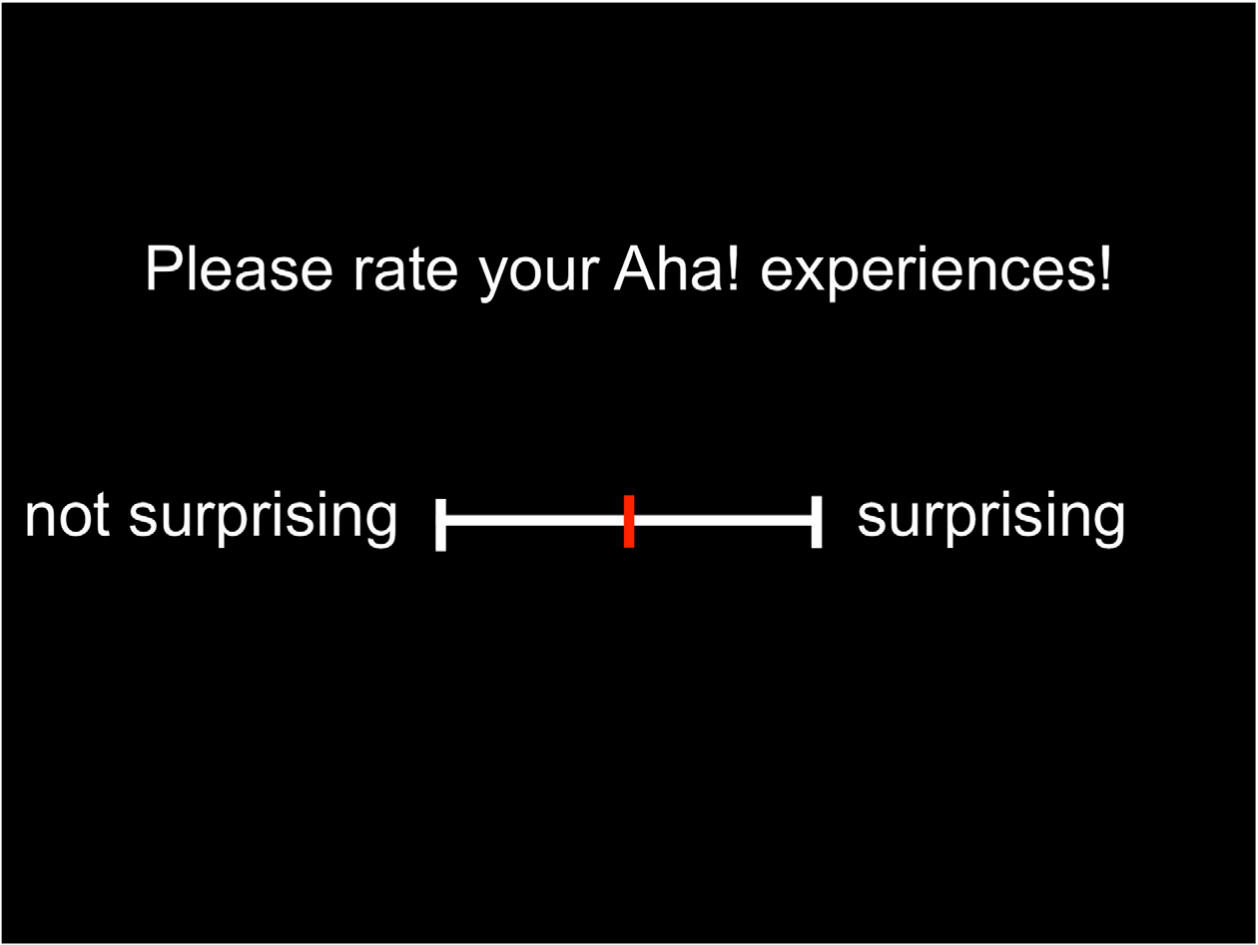
**Visual analog scale for the dimension surprise**.

***Session 1: rating of importance***. Subsequently, participants rated their Aha! experiences on each dimension separately, using a visual analog scale. For each dimension, a new scale was displayed on the screen (see Figure 2), with specific text on top of the scale and specific end point denominations (translated from the German original for the purpose of this paper).

- Please rate your Aha! experiences! unpleasant—pleasant
- Please rate your Aha! experiences! not surprising—surprising
- The solution came to me… slowly—quickly
- I felt about the solution… uncertain—certain
- Before the Aha! moment I felt… in no impasse—in an impasse

These descriptions refer to the dimensions happiness, surprise, suddenness, certainty, and impasse. As default, the cursor was set in the middle of the scale and participants moved it along the scale using the mouse to select a position. The left end of the scale corresponded to a value of 0 and the right end to a value of 100, but participants did not see any numbers. Participants were instructed as follows: “Think back to the Aha! moments that you had during the experiment. Now we ask you to rate them with regard to different aspects. Please indicate on the scale how much each aspect applies to your Aha! moments.”

To control for familiarity, at the end of session 1 participants received a questionnaire with a screenshot from each trick and were asked to indicate whether the solution of a trick had previously been known to them. These tricks were excluded on an individual level and handled as missing values (5.2% of all trials).

#### Session 2: re-assessment of Aha! experience

To control for its stability across time, the same assessment (self-report and rating of importance) was conducted 14 days later. The procedure was identical to session 1. Again, participants were explicitly asked to refer to the Aha! experiences they had had during the experiment (now 2 weeks ago) and to describe them from memory.

## Results

### Response coding and categorization of self-reports

Participants' solutions were coded off-line as true or false by two independent raters, Cronbach's alpha as a measure of inter-rater reliability was 0.99. True solutions were identical with the procedure that the magician had actually used. False solutions consisted of methods that were impossible with respect to the conditions seen in the video clip. If no solution at all had been suggested, the tricks were coded as unsolved.

Each participant produced a free report of their subjective Aha! experiences that was repeated after a 14 day delay. For six participants, the second rating was missing. The full statements are provided as Supplementary Material (translated from German). The reports were sorted into five main categories (see below). To avoid any a priori assumptions about the nature of Aha! experiences, the categories were compiled by a rater who was blind to the experimental rationale, and who based the compilation only on data from session 1. The rater read all statements from session 1 and collapsed them into meaningful, self-created categories. This rating scheme was subsequently used by three independent raters who re-categorized all reports (both session 1 and 2). A categorization was valid if at least two of the three raters assigned the same category. Critical ones were discussed until an agreement was reached. Each report could be assigned to more than one category, because participants often mentioned several different aspects that belonged to different categories. These were the categories:

1. Cognitive aspects
  - Elaboration (compare representational change theory, Ohlsson, 1992): A solution is found because a crucial detail is detected. This means, the initial problem representation is enriched with additional, previously overlooked details that eventually lead to a solution.
  - Restructuring (compare Ohlsson, 1992): A new way of looking at the problem, separate parts suddenly fit together, everything falls into place.
2. Emotional aspects
  - Happiness: feelings of joy, contentment, pleasure, positive arousal.
  - Tension release: strain is released, feelings of relaxation and relief.
  - Performance-related emotions: pride, drive, increased motivation, competitiveness, satisfaction.
3. Somatic reactions: physiological arousal or other reactions related to the body.
4. Reproduction of instruction: if participants simply repeated or paraphrased parts of the instruction that described the “standard” Aha! experience, this category was assigned, including the following aspects: Suddenness, rapidness, clarity of solution, certainty about correctness of solution, light bulb metaphor and common conceptions of Aha! experiences (e.g., “struck by lightning, the penny has dropped”).
5. Other: rest category

### Magic tricks

Table 1 provides an overview of the problem solving data obtained in session 1. See Danek et al. (2014) for a detailed analysis of solution rates, solution accuracy, certainty and influence of demographic variables.

**Table 1:**
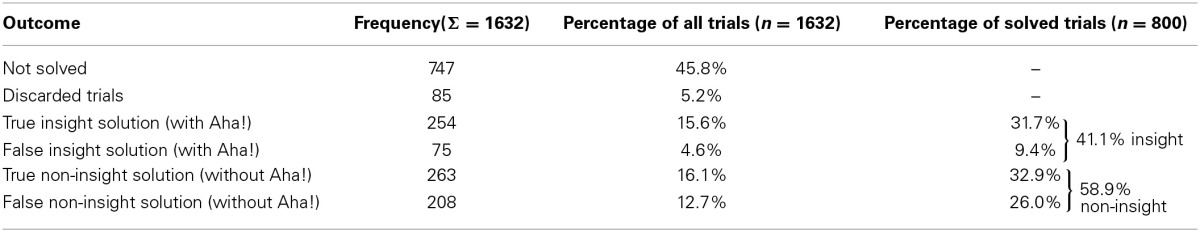
**Solution rates collapsed into different categories**.

*Thirty-four tricks × Forty-eight participants yielded a total of 1632 trials. Fifty-one % of them were either not solved or discarded due to familiarity of the trick (see first two rows) and 49% of all trials were solved (see four last rows). False solutions refer to implausible or even physically impossible solution suggestions*.

For 41% of all solved magic tricks, participants indicated that they had experienced an Aha! during the solving process. Of course, the subsequent Aha! assessment referred only to those events. Participants had been instructed to think back to their insight experiences, and to rate only those (compare methods).

### Assessment of Aha! experience

#### Reliability of Aha! ratings across time (ratings of importance)

There was a delay of 14 days between the first and the second rating time point. We addressed the reliability of those ratings by statistically comparing the two time points. For six participants, the second rating was missing.

**Figure 3:**
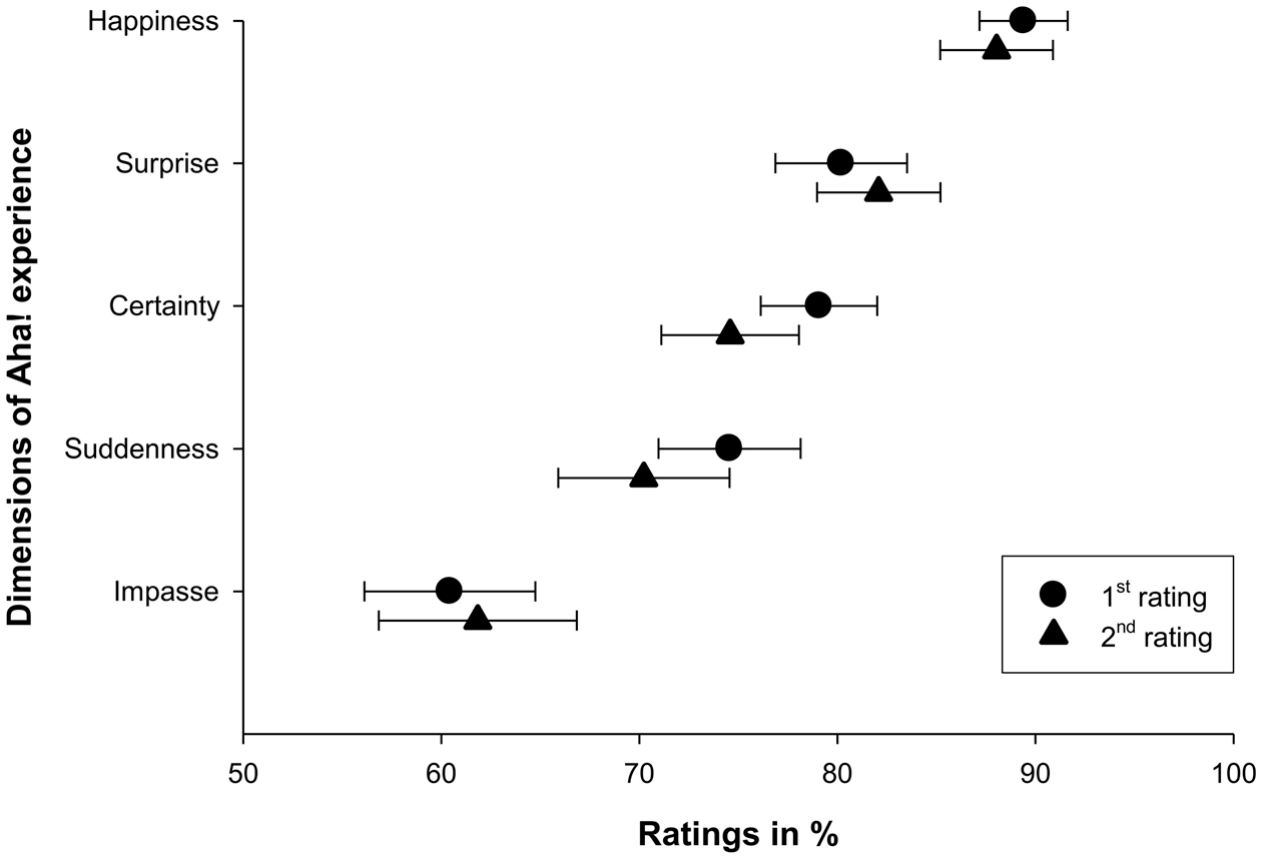
**Comparison of the averaged 1st (circle) and 2nd (triangle) importance rating for each dimension**. For each time point, the mean rating across participants is depicted. Horizontal bars denote standard errors of the mean.

Figure 3 shows that the 2nd rating of importance (conducted in session 2) did not differ substantially from the 1st rating (session 1). This observation was confirmed by a repeated measures ANOVA with the factors Session (two levels: session 1 and session 2) and Dimension (five levels: suddenness, surprise, happiness, impasse and certainty) that revealed no significant main effect for the factor Session [*F*_(1, 41)_ = 1.1, *p* = 0.3]. Thus, participants' ratings of their Aha! solution experiences remained stable across time.

There was a significant main effect for the factor Dimension, *F*_(4, 164)_ = 16.43, *p* < 0.01, indicating that there were differences between dimensions. We will focus on the two dimensions that significantly differed from all others, the one with the highest (happiness) and the lowest (impasse) rating, respectively. Pair-wise post hoc comparisons revealed that happiness (mean 88.5%) was rated significantly higher than all other dimensions (all *p* < 0.05). This means, happiness was the most important aspect of the Aha! experience. The feeling of being stuck in an impasse was in comparison less often reported: Impasse ratings were in general lower (mean 60.9%), and differed significantly from all other dimensions (all *p* < 0.05).

#### Analysis of self-reports

Table 2 shows how often the aspects had been described and provides one prototypical example each.

**Table 2:** **Categorization of participants' self-reports with prototypical examples (translated from German)**.

**#**	**Category**	**Example**	**Frequency in session 1**	**Frequency in session 2**	**Total frequency**
1a	Cognitive (elaboration)	I detected a small detail and suddenly, the things that I had observed previously make sense.	8	1	9
1b	Cognitive (restructuring)	What in the beginning didn't fit together suddenly makes sense.	6	2	8
2a	Emotional (happiness)	I am happy and get into a good mood.	20	23	43
2b	Emotional (tension release)	I feel relieved and relaxed.	8	11	19
2c	Emotional (performance-related emotions)	- I was much more motivated to continue working on the task. - Like a competition between me and the magician, and in Aha! moments, I felt like the winner. - I feel so much more intelligent.	12	12	24
3	Somatic reactions	Like a shot through my body.	3	3	6
4	Reproduction of instruction	I suddenly feel an enlightenment.	29	22	51
5	Other		6	4	10
			Σ 92	Σ 78	Σ 170

*Their corresponding frequencies are listed separately for the two time points, as well as summed up (last column)*.

For the 1st assessment (from session 1), comparing the cognitive and the emotional categories (1a + 1b vs. 2a + 2b + 2c) with a cross tab, we found that 24 participants mentioned emotional aspects (but no cognitive ones) whereas only 5 participants mentioned cognitive aspects (but no emotional ones). This difference was significant (McNemar test, *p* < 0.01).

After 2 weeks, this difference was even more pronounced: In session 2, 30 participants mentioned emotional, but no cognitive aspects (in contrast to only two participants with the reverse pattern), and the McNemar test was significant with *p* < 0.01.

Regarding the emotional categories, clearly the most relevant aspect was happiness (mentioned 43 times). Performance-related emotions (24 times) and the feeling of tension release (19 times) were mentioned less often.

Apart from reproductions of the instruction, which dealt mainly with the solution strategy used (Aha! vs. more analytic solving styles), only few cognitive aspects were mentioned.

Somatic reactions were only mentioned by three participants at each time point. Two statements were from the same participants, i.e., in session 2, two participants described the same physiological reactions as they had during the first session. In the first case, this was “a slight pull in my chest and tummy,” and the second participant expressed the feeling “like a shot through my body.”

Category 4 was used as a manipulation check. Obviously, participants remembered the instruction well or used the same characteristics, with 51 total instances of naming one of these aspects.

## Discussion

The new task domain of magic tricks proved to be well suited to trigger Aha! experiences with 41% of all solutions classified as such. This finding provides further evidence for our conception of magic tricks as an insight task (see Danek et al., 2014). The comprehensive assessment of solution experiences revealed participants' strong emotional involvement upon gaining insight into the working of a magic trick. To our knowledge, this emotional component of insight has not been specifically documented yet for any other problem solving task. We therefore advocate magic tricks as useful tools to investigate insight problem solving.

With regard to phenomenology, the present results support our conception of the Aha! experience as multi-dimensional. However, the hypothesis that all five dimensions of the Aha! experience would be rated as equally important was not confirmed. Instead, we found “happiness” as prevailing aspect. This primacy of positive emotions is also reflected in participants' self-reports although two different methods were used (qualitative self-reports and quantitative ratings on a visual analog scale with fixed dimensions).

The dimension “impasse” appears to be less important than previously thought (Ohlsson, 1992), casting doubt on the theoretical assumption that being in a state of impasse is a prerequisite for experiencing insight later. This finding is in accordance with results from a study on the Candle Problem (Duncker, 1945) by Fleck et al. (Fleck and Weisberg, 2004) who found only few instances of impasse in verbal protocols obtained during the solution process. However, this finding might perhaps also be attributed to characteristics of our new stimulus domain. We argue that watching a magic trick directly puts the observer in a state of impasse—namely in the first moment of astonishment and wonder about the magic effect. At first, the observer is left completely baffled, without any solution prospect. But later, after the problem solving process has been initiated, participants don't necessarily experience an impasse.

The importance ratings remained stable across time in all five dimensions (see Figure 2). To evaluate such a fleeting moment by pinpointing its dimensions on five different scales is arguably quite a difficult task. It seems impressive that participants were able to recall their Aha! experience so vividly after 14 days that they rated it identically. This finding provides empirical support for Bowden's claim (2005) for the usefulness and reliability of self-reports in insight research.

A weakness of the visual analog scale used here is the lack of negatively poled questions, reflected in the answers' general trend toward the positive pole. The temporal stability of the importance ratings might thus partly be explained by reduced variability caused by this positive bias. An alternative explanation for the ratings' stability must also be considered: It is conceivable that participants did not actually remember their Aha! experiences, but instead reported what they remembered reporting in session 1. However, this seems unlikely for two reasons: First, participants had not been informed about what would happen in the second experimental session—they were completely unaware that the rating would have to be repeated. Second, to make it difficult to remember the previous rating, we had deliberately implemented a visual analog scale without any numbers. There was only a line on which the cursor had to be positioned. In this way, participants could never know the value to which the selected position corresponded and could therefore not retain any numbers, only a visual image of the scale. It seems unlikely that participants were able to incidentally retain this visual impression for 2 weeks for five dimensions, especially when considering the complex wording of the different rating scales (see Section Session 1: rating of importance).

The free self-reports helped to obtain further information about problem solvers' actual experience. A qualitative analysis of this data revealed positive emotions as the prevailing aspect of Aha! experiences. These quotes from two of our participants may serve as an illustration: “A moment of bliss. I am happy and get into a good mood.” and “Explosively, the bad feeling of frustration and confusion turns into a feeling of happiness and I feel a swell of pride.” (see Supplementary Material). This is in accordance with results from the importance ratings in which happiness received the highest value. We thus demonstrated the occurrence of strong positive emotions during sudden moments of insight.

We found two new aspects in participants' self-reports. The comparably high frequency of performance-related aspects (e.g., “I feel really clever now” or “With Aha! experiences, I am much more motivated to continue working on the task or problem”) has not been reported before. However, it can be assumed that this aspect is relevant for many problem solving tasks since participants' cognitive abilities are put to the test. Finding a solution can be experienced either positively or negatively (chagrin about prior “stupidity,” compare Gick and Lockhart, 1995). The present data suggests that the majority of participants felt happy about being able to solve the magic trick, see above. That some participants felt a heightened motivation to continue with the task after an Aha! experience offers many possibilities for interesting follow-up studies. For example, Aha! experiences could be used to motivate students in classroom settings.

Although we subsumed them both under the category “performance-related aspects,” the comments about motivation and cognitive abilities must be differentiated from comments about a competition with the magician (e.g., “The magician can't fool me anymore because by now, I could do the trick by myself”). This was not expected, and at first glance, might be attributed to the special task situation with our participants being confronted with the magician as a kind of rival, thus engaging in a competition with him. However, even if no direct opponent is presented, a certain flavor of competitiveness is a shared characteristic of all problem solving experiments. Typically, participants are worried that their level of intelligence will be assessed or that the experimenter will find out that they perform worse compared to other participants. Thus, they either compete against the experimenter (who typically knows all the solutions) or against other participants. Consequently, if our comprehensive assessment of Aha! experiences would be conducted with traditional problem solving tasks, we would expect similar results. Of course, this remains to be shown in future studies.

Tension release was the other new aspect of the Aha! experience (e.g., “I feel relieved and relaxed now” or “feeling of relief after a phase of strain caused by failure”). It seems plausible to assume that tension arises if there exists no obvious solution for a problem. During unsuccessful problem solving attempts, the tension builds up further. If at last, unexpectedly, a solution is found, the tension will rapidly decline. Apparently, this is an important aspect still missing from current definitions of the Aha! experience.

These empirical findings relate to theoretical assumptions about the phenomenology of the Aha! experience. Ohlsson (1984) summarized the Gestalt psychologists' main ideas in a set of principles. Some of them overlap with the self-report data: In the category “performance-related emotions,” participants repeatedly describe heightened motivation (“I am much more motivated to continue working on the task”). This closely resembles proposition N (Ohlsson, 1984, p. 70) in which an “energizing effect on problem solving behavior” is described. Other aspects also match: “Recentering as a displacement of attention from one part of the situation to another [… ] reveals what the central part of the situation really is” (Ohlsson, 1984, p. 70). This corresponds to the “elaboration” category and matches the idea of selective encoding (Davidson, 1995), i.e., that a problem solver suddenly detects certain features which were not obvious before (and not encoded) as relevant for a solution. For example, one of our participants noted that “Through a small detail, the entire action sequence becomes clear.”

We conclude that there is a wealth of information to be gained through subjective self-reports. Most participants took several minutes to diligently describe their thoughts, using vivid and expressive language as documented in the Supplementary Material. We recommend the use of such direct, qualitative self-reports as a promising tool to learn more about the phenomenological aspects of insight problem solving.

Of course, there are obvious limitations to the introspective method: It is highly subjective, and general conclusions can only be drawn with caution. Moreover, it is difficult to clearly determine what participants actually used as the basis for their report, especially if several defining aspects of the experience in question are mentioned in the instruction, as done in the present study. Durso even suggested that because participants were shown to be unable to correctly judge their progress toward a solution (Metcalfe, 1986), “… self-reports following insight are equally unreliable.” (Durso et al., 1994, p. 94). Yet we argue that for the elusive phenomenon of insight, subjective Aha! reports might provide information that would not be accessible through more rigorous experimental methods. Other researchers have successfully used verbal protocols to elucidate the processes during insight problem solving (Kaplan and Simon, 1990; Dominowski and Buyer, 2000; Fleck and Weisberg, 2004; see also Fox et al., 2011, for a recent meta-analysis on verbalization procedures in general) and others even argue that the rejection of introspective methods hinders the advancement of the field (Jäkel and Schreiber, 2013). We suggest that the traditional approach of using pre-defined “insight problems” and assuming the occurrence of insight in the case of a solved problem, without taking into account participants' individual problem solving experiences, should always be complemented by subjective measures (e.g., Aha! self-reports or thinking-out-loud protocols) directly obtained from participants.

Another limitation of the present study is that we did not collect any ratings on non-insight solutions. On a trial-by-trial basis, additional ratings would have increased task demands too much (considering the large number of difficult problems that participants had to solve). But a second global rating at the end for non-insight solutions, too, would have been feasible. This would have offered the possibility of directly comparing the two types of solutions and thus would have allowed answering questions regarding the difference in participants' subjective experiences while solving problems with or without insight. Future studies should incorporate this improved design. However, since the focus of the present study was on the phenomenology of the Aha! experience, aiming to disentangle its several components, we decided not to introduce any ratings on non-insight solutions. Instead, participants concentrated on insight solutions in all ratings, with the aim of grasping the Aha! experience as fully as possible.

Critical appraisal of magic tricks as problem solving tasks: We claimed that magic tricks represent a more authentic task domain than previous insight tasks because participants start working on the problem quite naturally, eager to find out the magician's secret. During the testing, participants were highly motivated to solve the presented tricks, even after many trials. In addition, magic tricks are less artificially construed than classical insight problems in which participants must solve verbal riddles, logical brainteasers, mathematical problems or connect dots according to arbitrary rules. They are authentic because they take place in familiar situations with ordinary objects like coins or cigarettes. The present work indicates that such authentic stimuli can be as valuable as strictly controlled paper-and-pencil tasks. A systematic comparison of magic tricks with traditional types of stimuli (e.g., with regard to motivational aspects) is needed to further substantiate this claim.

Another open question is how much the findings from the present study about insight in a magic context will generalize to other tasks. It is actually a weakness of most problem solving studies, ours included, that only one type of task is used (but there are exceptions, e.g., Metcalfe and Wiebe, 1987). Attempts at setting up taxonomies of “insight problems” show the large range and heterogeneity of tasks used (Weisberg, 1995). Future studies should include different types of problems to allow a direct comparison of the results across tasks. However, we are confident to assume that the present findings will generalize to other insight problems, because, applying the framework of the representational change theory (Ohlsson, 1992), it seems obvious that classical insight problems and magic tricks rely on fairly similar processes. Both activate self-imposed and over-constrained problem representations that have to be relaxed in order to come up with a solution. Our rationale for using magic tricks as an insight task is explained in detail in Danek et al. (2014). Moreover, we could already show (Danek et al., 2013) that magic tricks that are solved by insight had a higher recall rate after 2 weeks, a similar effect as found with classical insight problems.

Inducing positive mood could be another important advantage of using magic tricks in insight research, because it has been shown previously that positive affect facilitates insight (Isen et al., 1987; Bolte et al., 2003; Subramaniam et al., 2009; Sakaki and Niki, 2011). For example, Isen et al. (1987) induced positive mood by presenting a comedy film (Gag reel) to participants shortly before they began working on Duncker's Candle Problem (1935). A control group who had watched a neutral film (a math film, Area under a curve) produced significantly less solutions than the positive mood group. It seems plausible that in the present study, participants' emotional state was positively influenced by watching the magic tricks, similar to watching a comedy film. The self-reports showed the high emotional impact of solving a magic trick. Although we did not directly assess mood, it was obvious that participants liked to watch the tricks and were highly motivated to do the task. Perhaps the drop-out rate of zero (for the second visit to the lab) can also be accounted to that. In pilot studies, participants scored very high on the question “How much did you like the trick?” with a mean of 2.94 (on a rating scale from 1 = not at all to 4 = very much). We speculate that the positive mood induced by watching magic tricks also facilitated insight in the present study. In future experiments using magic tricks, we recommend to systematically control for mood.

In sum, this study demonstrates that the Aha! experience should not only be regarded as an interesting epiphenomenon or trial-sorting criterion, but that the phenomenon itself can be investigated systematically and fruitfully. Implementing magic tricks as problem solving task, we could show that strong Aha! experiences can be triggered if a trick is solved. We could at least partially capture the phenomenology of Aha! by identifying one prevailing aspect (positive emotions), a new aspect (release of tension upon gaining insight into a magic trick) and one less important aspect (impasse). We hope to have contributed to a deeper understanding of the nature of this complex phenomenon by introducing magic tricks as a useful research tool for insight problem solving.

### Conflict of interest statement

The authors declare that the research was conducted in the absence of any commercial or financial relationships that could be construed as a potential conflict of interest.
