# Supplementary material for: It's a kind of magic—what self-reports can reveal about the phenomenology of insight problem solving
Source: Front Psychol. 2014 Dec 8;5:1408. doi: 10.3389/fpsyg.2014.01408 (PMC4258999; doi:10.3389/fpsyg.2014.01408)
Supplement: Supplementary file 1 [file DataSheet1.DOCX]

1. **Supplementary Material**

**Self-reports (participant numbers are listed)**

1. A moment of bliss. I am happy and get into a good mood. An increasing certainty. Everything becomes perfectly clear, a tingling in my head.

2. I’m excited and I feel no doubt about this sudden solution.

3. A sudden discovery, unexpected, a feeling of "that's how the solution must be", no hesitation

4. A feeling of definite knowledge or alternatively, a first sensation of knowledge that is not necessarily confirmed in the next step, but initially, feels certain and irrefutable. A sense of triumph because one has seen through the trick. "Gotcha!"-feeling. Yes, of course, there is no other way! As long as any doubts about the correctness remain, it is no Aha! effect.

5. I perceive a certain movement of the hand, from which I can infer how the trick could possibly work. Thus, it can only be detected at a certain time point of the trick. Classic Aha! experience, a feeling of seeing through the trick all at once.

6. For me, I experienced an Aha! either when I could see the solution in my mind or when I had the feeling of knowing the solution. This feeling is similar to being motivated to do something, knowing that it is exactly the right thing. With Aha! experiences, I am much more motivated to continue working on the task or problem.

7. I did not experience any Aha!

8. Sometimes it was a very strong feeling, as if I had solved an extremely difficult case that mattered greatly. Other times, it was more like "ok, now I got you and I know how you did it". In the first case, the excitement was much greater, in the second case, it was more relieving.

9. It can perhaps be described as a flash, suddenly I knew the answer, even if shortly before, I didn't have a clue. It's a wonderful, positive feeling and for me, it felt a bit like relief.

10. It's like a small tension that gets released suddenly and a positive and liberating feeling emerges.

11. Suddenly, without prior warning, the only plausible solution pops out in my mind. Feeling of joy.

12. In a split second, I'm struck by a flash of genius.

13. When suddenly the brain knows how it happened.

14. Very good. I believe that the magician can't fool me anymore because by now, I could do the trick by myself. With an Aha! experience, I feel very sure about the solution.

15. Suddenly, everything becomes perfectly clear, the missing link is found. It is awesome to suddenly see through the trick, because I feel very clever. It just clicks and it is a very positive feeling. Like a reward for thinking so hard. I feel lively and happy to have figured it out. A feeling of bliss.

16. When the different parts fall into place and my considerations make sense. A slight pull in the chest.

17. It‘s like a shot through my body. Being awakened from previous ignorance, I feel really happy.

18. Explosively, the bad feeling of frustration and confusion turns into a feeling of happiness and I feel a swell of pride.

19. I detected a small detail and suddenly, the things that I had observed previously make sense. It feels like the penny has dropped, and I feel a bit proud to be so certain now, although I had no clue just a few seconds before.

20. I feel that suddenly, I know the solution, thrilled, excited, pleased to have understood something.

21. It seems as if in this moment, all confusion in my brain becomes resolved and I should have known it earlier, simply because it is so logical. The Aha! experience can really be described as the feeling of switching on a light bulb. And I feel somehow affirmated and positively relaxed.

22. I had no Aha! experiences.

23. Abruptly, it becomes clear what's hidden behind all this. When I search for a solution, and suddenly the tension gets released, because I figured it out. A release of tension occurs in my head.

24. „It dawned on me“ is a saying which can very well be connected with this effect; you have thought about a certain matter for a while and suddenly, there is a detail you had not noted earlier which now is leading you in a different direction. It’s also a moment of relief and relaxation – time seems to have come to a standstill for a short while. Maybe it’s some sort of very short flow experience.

25. In contrast to the other moments I didn’t have to check the realisation; the solution seemed to be unambiguous, mostly like remembering something. Usually I’m quite certain about it.

26. Suddenly one knows or believes to know how it works without checking it in detail.

27. The moment comes quite suddenly, as if the idea jumps directly into your mind and doesn’t develop step by step by reflection. I am happy about the surprising knowledge.

28. A sudden image appears before my inner eye, triggering off an impulse for action. The further course of the magic trick becomes uninteresting, the focus is on the image which seems to be the solution or actually is the solution.

29. You can’t understand why you didn’t see that before although it is so simple. It didn’t come about by logical reflections but rather simply because it has to be as it is.

30. Like a sudden relief after a time of tension, a feeling of happiness. What in the beginning didn’t fit together suddenly makes sense. Thoughts can keep flowing where before they were in front of a barrier.

31. The sudden understanding of the solution. The solution becomes visible before my inner eye in a flash.

32. During an Aha! experience you suddenly realize a detail; somehow you are happy to have seen through the magic trick.

33. Satisfaction at having found the correct solution.

34. With an Aha! experience I suddenly feel an enlightenment; it is as if quite suddenly the light was switched on or a switch turned.

35. Very quick feeling, easy and liberating.

36. I think the magician always did something to distract me from his tricks. So, after the second viewing, I concentrated on what had changed or had disappeared. As soon as I did so, I had this Aha! experience and I was happy.

37. Very happy and surprising.

38. An Aha! experience feels good. Here, once in a while, suspicious hand movements of the magician were interpreted as important for a sudden understanding of the trick; other Aha! experiences felt like a sudden memory of a similiar trick. Aha! experiences felt like ideas, similar to agreeable memories suddenly having come back.

39. Beautiful, surprised by oneself.

40. Can’t remember exactly, but it feels a bit like a sudden intuition and like „Oh, that’s it! Why didn’t I find out earlier?“

41. The Aha! experience is marked by a feeling of joy and personal satisfaction triggered off by finding the solution.

42. Through a small detail, e.g. the cut-off upper part of the spoon, the entire action sequence becomes clear, respectively foreseeable and further details can be detected, e.g. that the magician once again puts the spoon in his hand and quickly takes it out again.

43. Rather nice…you observe what you can see and try to explain how it works and then a decisive detail strikes you – suddenly you have an idea how it could work. As if you had found the last missing part of the puzzle in order to reconstruct the rest of the picture.

44. It feels like an enlightenment, a sudden inspiration how the problem can be solved. Suddenly all pieces of information fit together which before had not fitted together.

45. It feels good because suddenly I know something. It feels alright and, in a way, makes me happy.

46. Aha! experiences could be compared to a quick jump in my head. I didn’t have to think rationally about how the trick worked, but it simply came to me.

47. I feel as if a light had been switched on in my head and I’m thinking of my Latin teacher who kept speaking about this. We had lots of Aha! experiences with him.

48. A click in my head, as if someone had turned a switch but, nevertheless, very relaxing and I feel so much more intelligent.

**Self-reports (14 days later)**

1.-5. missing

6. Aha! experience – a short inspiration how to get out of this hopeless situation. This feeling gives me wings that make me continue working on the problem which I had not been able to solve before. And, naturally, I immediately feel inclined to solve further problems, as it seems you now can do anything, no matter which task you have been set.

7. No Aha! experiences.

8. I had two different kinds of Aha! experiences. With one, I felt like suddenly having an idea how the trick had been done – sort of thunder-struck! With the other Aha! experience, I felt as if I had reflected for a moment, I was rather relieved to have seen through the trick, whereas with the first kind I felt as if I had tricked the magician himself by understanding his trick.

9. I found the solution rather fast, I didn’t have to think about it at all and I had no clues from which to start thinking. There was a feeling of relief connected with the Aha! experience, it was a very positive feeling.

10. Some sort of tension is released, it is a relaxing, positive sensation and it also gives you some kind of feeling of success.

11. I was excited, felt good and satisfied, it felt as if the solution had suddenly appeared in my head.

12. The Aha! experience came suddenly and unexpectedly.

13. It suddenly appears in my head how the trick works and it somehow feels great to find this out, well, the feelings are: gladness, joy to have found out and curiosity, as you can’t always be sure if it really works like that.

14. Very good, I think I know exactly what has just happened. I have exactly understood the trick or the situation, I am quite sure to be right.

15. It simply goes snap und you’ve got it! Before it happens you keep thinking about it and don’t quite get round it, but when the Aha! experience occurs, then you suddenly understand everything – it’s like an insight into the whole situation.

16. Like a slight pull in my chest and tummy.

17. Excitement and a feeling like a warm swirl going through my body. I feel relieved and relaxed, joyful.

18. A sudden change from a feeling of insecurity and fear of failure to a feeling of joy and pride. Especially in this moment of change, I’m experiencing an incredible energy flowing through my body.

19. I am glad and proud to have found a solution, relieved, for a short while I forget what is happening around me.

20. I feel surprised that I have understood something, I am content, maybe even proud of myself, I feel pretty good and agreeable. It certainly is a positive feeling, making me more enthusiastic and full of energy.

21. An Aha! experience feels good, as if I actually saw a light switched on, suddenly everything appears to be quite clear.

22. I had none.

23. Like a flash of genius, as if suddenly everything became clear, you keep brooding and then it suddenly appears, tension disappears, you feel released, a bit euphoric.

24. As if a light was switched on, something that before I hadn’t properly paid attention to now turned out to be the cause of a whole series of effects.

25. Sudden remembrance, dissatisfaction about not having discovered it earlier.

26. Suddenly I know the answer, it is as if a light lightened up the darkness.

27. It is as if the idea appeared suddenly out of nowhere and came into my mind. I’m glad about the surprising knowledge and I’m very content.

28. Suddenly an image appears in my mind’s eye, everything else around me becomes uninteresting and the concentration is focused only on that image which could be, respectively is, the solution of a problem.

29. Strange that I haven’t seen the solution earlier, it turned out to be much easier than I would have expected. At some point, the understanding is simply there.

30. It was a feeling of relief combined with a feeling of happiness after a phase of strain caused by failure.

31. A sudden moment, a feeling of understanding as if it began to dawn on me.

32. It felt good to have found the solution. It was like a competition between me and the magician, and in an Aha! moments, I felt like the winner.

33. When I discovered the solution, I sudddenly was content about myself.

34. It is like a sudden flash of insight, as if someone had switched on a lamp bringing light into the darkness. This moment comes as a surprise and without warning.

35. A slight feeling as if something fell off. For a short moment, you get a feeling of a clear sight.

36. With some tricks, the understanding came suddenly, very easy and I felt glad.

37. Surprising and funny.

38. You have an Aha! experience when understanding connections, thus creating a meaningful image. It can be compared to the good feeling of having finished something that had cost you a lot of time and effort, a positive feeling of understanding.

39. Beautiful. Surprised. Fulfillment of my ambitions. Cool.

40. A moment when you think: Ah, that’s it! Why didn’t I see that before!

41. An Aha! experience is very satisfying, you think you have finally understood how it works and that you have unmasked the trick. Also in other situations, Aha! experiences feel like that. Immediately, you are completely sure about it.

42. Suddenly the solution appears quickly in my mind. In most cases, it is quite a different track than I had expected before. All the other steps simply follow.

43. I have a smaller or greater experience of success when I believe to have understood the trick.

44. A problem is suddenly understood and solved, it’s like a stroke of genius and sometimes you don’t even know how you found the solution.

45. A feeling of great satisfaction, a redeeming, relaxing moment, making me feel happy and satisfied.

46. A moment that liberates your mind, relieving your mind from strain, you feel enlightened.

47. I was ever so pleased about having seen through the trick, respectively a little less pleased.

48. A relaxing feeling, feels good and at the same time you don’t feel as excited as before.
